# Multicenter, randomized controlled trial of EUS-guided fine-needle biopsy using a fork-tip needle with macroscopic or rapid on-site evaluation for pancreatic lesions (H_2_O trial)

**DOI:** 10.1097/eus.0000000000000087

**Published:** 2024-11-08

**Authors:** Takeshi Ogura, Susumu Hijioka, Kazuo Hara, Nobu Nishioka, Atsushi Okuda, Saori Ueno, Hiroki Nishikawa, Masanori Yamada, Yoshikuni Nagashio, Yuya Hisada, Yumi Murashima, Kotaro Takeshita, Shin Haba, Takamichi Kuwahara, Nozomi Okuno

**Affiliations:** 1 Endoscopy Center, Osaka Medical and Pharmaceutical University, Osaka, Japan; 2 2nd Department of Internal Medicine, Osaka Medical and Pharmaceutical University, Osaka, Japan; 3 Department of Hepatobiliary and Pancreatic Oncology, National Cancer Center Hospital, Tokyo, Japan; 4 Department of Gastroenterology, Aichi Cancer Center, Nagoya, Japan; 5 Department of Gastroenterology and Hepatology, National Cancer Center for Global Health and Medicine, Tokyo, Japan; 6 Department of Gastroenterology, Matsusaka Chuo General Hospital, Matsusaka, Japan; 7 Department of Gastroenterology, Tane General Hospital, Osaka, Japan.

**Keywords:** EUS, EUS-FNA, EUS-guided fine-needle biopsy, Rapid on-site evaluation, Macroscopic on-site evaluation

## Abstract

**Background and Objectives:**

According to previous reports, EUS–fine-needle biopsy (FNB) with or without rapid on-site evaluation (ROSE) showed the nonsuperiority of EUS-FNB + ROSE over EUS-FNB. However, previous studies included various kinds of FNB needle. This might be a critical limitation due to heterogeneity. Therefore, the aim of the present multicenter, randomized controlled trial was to compare the diagnostic accuracy of the fork-tip needle with or without ROSE for pancreatic lesions.

**Methods:**

In the ROSE group, if an adequate sample was obtained to diagnose by on-site evaluation, EUS-FNB was stopped. If cytological results were insufficient or indeterminate, EUS-FNB was repeated. In the macroscopic on-site evaluation (MOSE) group, if a 4-mm length of visible core tissue was obtained, EUS-FNB was finished. If visible core tissue was not obtained or was less than 4 mm in length, a second puncture was attempted.

**Results:**

One hundred seventy-one patients were randomized, 85 to the ROSE group and 86 to the MOSE group. In the MOSE group, diagnostic sensitivity and accuracy were 94.4% and 91.8%, respectively, for visible core tissue and 80.6% and 70.0%, respectively, for red tissue. Finally, overall diagnostic sensitivity and accuracy were 97.1% and 95.3%, respectively, in the ROSE group and 95.8% and 95.3%, respectively, in the MOSE group. Although there were no significant differences regarding adverse events between groups, the mean number of punctures was significantly lower in the MOSE group than in the ROSE group (1.47 *vs*. 1.20, *P* = 0.0171).

**Conclusions:**

EUS-FNB using a fork-tip needle for pancreatic lesions has high diagnostic yield even without ROSE.

## INTRODUCTION

EUS-FNA is a well-established technique to obtain cytological confirmation for pancreatic lesions.^[1]^ To improve its diagnostic yield, EUS-FNA combined with ROSE has been reported.^[2,3]^ ROSE has several benefits, such as providing timely feedback on sample adequacy and optimizing the number of needle passes required to obtain a diagnosis during EUS-FNA. Indeed, in a meta-analysis of EUS-FNA combined with ROSE, ROSE was associated with up to a 3.5% improvement in adequacy rates for EUS-FNA of solid pancreatic lesions.^[4]^ On the other hand, to obtain histological specimens, various FNB devices, such as the side-fenestrated, forward-facing bevel needle (ProCore; Cook Medical, Bloomington, IN), the fork-tip needle (SharkCore; Medtronic, Medtronic Corp., Boston, Mass), and the Franseen tip needle (Acquire; Boston Scientific, Boston Scientific Corporation, Marlborough, Mass, USA), have recently become available. According to a randomized controlled trial,^[5,6]^ the diagnostic yield has been reported to be greater than 90%; therefore, FNB might play an important role for ancillary molecular analysis and personalized medicine in the future.^[7]^ Given this background, the roles of ROSE in clinical practice may not be clear. Indeed, recent studies^[8,9]^ and a meta-analysis^[10]^ of EUS-FNB with or without ROSE showed the nonsuperiority of EUS-FNB + ROSE over EUS-FNB with newer end-cutting needles. However, the previous randomized trials and meta-analysis included various kinds of FNB needle. This might be a critical limitation due to heterogeneity or inadequate evaluation of the diagnostic yield of EUS-FNB. Therefore, the aim of the present multicenter, randomized controlled trial was to compare the diagnostic accuracy of the fork-tip needle with or without ROSE for pancreatic lesions.

## METHODS

### Study design

This was a multicenter (Aichi Cancer Center Hospital, National Cancer Center Hospital, Osaka Medical and Pharmaceutical University Hospital), randomized, noninferiority trial comparing EUS-FNB with macroscopic on-site evaluation (MOSE) to EUS-FNB with ROSE in the diagnosis of pancreatic lesions. This trial was approved by the Certified Review Board of Osaka Medical and Pharmaceutical University Hospital (2020-015-4) and by the institutional review boards of all participating centers. All patients provided their written, informed consent. The trial was registered with the University Hospital Medical Information Network Clinical Trials Registry (UMIN000041676).

All consecutive patients with pancreatic lesions who presented to 3 referral hospitals for EUS-FNB between August 2020 and May 2022 were prospectively enrolled using a designated website. Using a web-based registration program, the included patients were randomized 1:1 to 2 groups in blocks of 4 stratified by sex (male or female), age (>70 or ≤70 years old), lesion size (>20 or ≤20 mm), location of lesion (head of pancreas or pancreatic body and tail), and hospital (Aichi Cancer Center Hospital, National Cancer Center Hospital, or Osaka Medical and Pharmaceutical University Hospital). One group underwent EUS-FNB with MOSE, and the other underwent EUS-FNB with ROSE.

### Eligibility criteria

The inclusion criteria were as follows: (1) pancreatic lesion detected by diagnostic imaging such as computed tomography; (2) at least 20 years of age; (3) accessible papilla; and (3) Eastern Cooperative Oncology Group performance status (ECOG PS) ≤2. The exclusion criteria were as follows: (1) blood vessels or other tumors between the lumen and the target lesion; (2) bleeding tendency, defined as international normalized ratio of the prothrombin time >1.5 or a platelet count <50,000 cells/mL; (3) surgically altered anatomy, or serious dysfunction in other organs; or (4) otherwise judged by the investigator to be ineligible for inclusion.

### Procedure protocols

**Figure 1:**
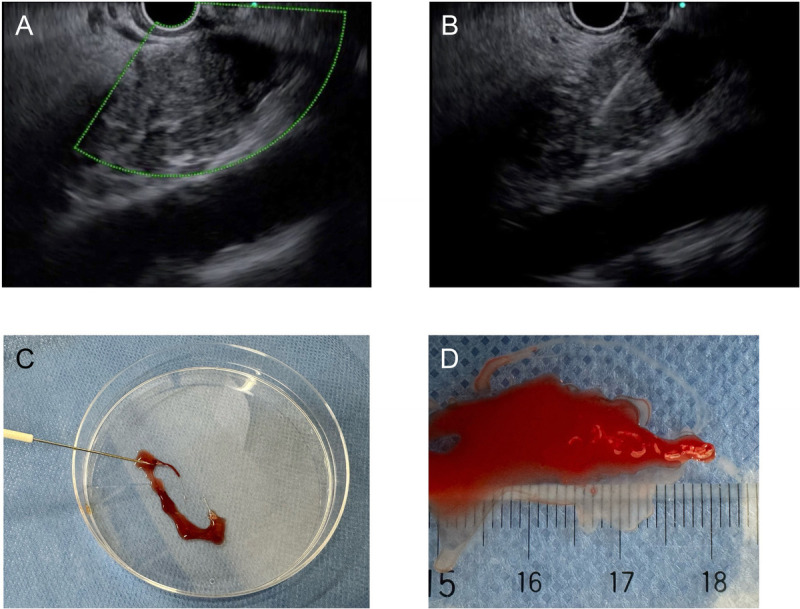
A, To avoid vessel injury, the puncture route is carefully evaluated using color Doppler mode. B, The needle is moved from the distal to the proximal sites of the lesion about 20 times. C, After needle removal, all materials are extruded from the needle into the Petri dish. D, Specimens are divided into visible core tissue and red tissue.

All procedures were performed by experienced endoscopists who had performed at least 100 EUS-FNBs before the start of this study. In this study, a 22-gauge fork-tip needle (SharkCore) was used. The echoendoscope (UCT260; Olympus Optical, Tokyo, Japan) was inserted into the gastrointestinal tract, and target lesions were then identified. To avoid vessel injury, the puncture route was carefully evaluated using color Doppler mode [Figure 1A]. The stylet was retracted approximately 5 mm, and puncture was attempted. In this study, EUS-FNB was performed using the slow-pull technique combined with funning technique, as previously described.^[11,12]^ The needle was moved from the distal to the proximal sites of the lesion about 20 times [Figure 1B], and the needle was then removed. After needle removal, all materials were extruded from the needle into the Petri dish [Figure 1C].

In the MOSE group, specimens were divided into visible core tissue and red tissue [Figure 1D]. The histological diagnostic yields of both tissues were evaluated independently. If a 4-mm length of visible core tissue, which might be considered core tissue of the pancreatic lesion, was obtained, EUS-FNB was finished. If visible core tissue was not obtained or was less than 4 mm in length, a second puncture was attempted. On the other hand, in the ROSE group, the tissue of interest was placed on a slide and then gently pressed down, rubbing with another slide and smeared by an experienced cytopathologist. This smear was stained with toluidine blue and evaluated for adequacy under the microscope. If an adequate sample was obtained to diagnose that it was malignant or benign by on-site evaluation, EUS-FNB was stopped. If cytological results were insufficient or indeterminate, EUS-FNB was repeated. All remaining tissue was used for histological evaluation.

### Follow-up and diagnosis

To evaluate the occurrence of all adverse events associated with the procedures, blood examination was performed the day after EUS-FNB, and all patients also underwent blood and physical examinations 7 days after EUS-FNB. The clinician who evaluated adverse events was not blinded to allocation to the ROSE or MOSE groups. Adverse events associated with EUS-FNB were evaluated according to the severity grading system of the American Society for Gastrointestinal Endoscopy lexicon.^[12]^

During ROSE, on-site results were divided into 3 categories: adequate (sufficient material for adequate cytological assessment), indeterminate (sufficient material for limited cytological interpretation), and insufficient material.^[13]^ Pancreatic lesions were diagnosed as malignant or benign. A final diagnosis was made using surgical specimens. In nonresection cases, the final diagnosis of malignancy for pancreatic lesions was made according to previously described criteria.^[14]^ Malignancy was considered present if the FNB results were either suggestive of malignancy or malignancy, and one of the following was present: (1) disease progression and/or development of metastases during follow-up, (2) stability or improvement of the lesion with chemotherapy, and/or (3) patient’s death or hospice care within 6 months of identification of the mass. In nonresection cases, the definition for a final diagnosis of benign disease was that FNB results were negative for malignancy, and one of the following was present: (1) follow-up imaging after >12 months reporting stability or improvement of the pancreatic lesion without receiving chemotherapy; and (2) cytological or histopathological diagnosis of benign disease with an appropriate clinical course of the disease for a minimum of 12 months. Serous cystic neoplasm, autoimmune pancreatitis, chronic pancreatitis, and neuroendocrine tumor (G1, G2) were considered benign, whereas pancreatic adenocarcinoma, neuroendocrine tumor (G3), and metastatic tumor were considered malignant.

### Outcomes

The primary outcome was the comparison of diagnostic accuracy, defined as the percentage of collected specimens matching the final diagnosis, between ROSE and MOSE. Secondary outcomes were diagnostic measures such as sensitivity, specificity, negative predictive value, positive predictive value, results of ROSE, number of needle passes, length of visible core tissue, and adverse events.

### Definitions and statistical analysis

Diagnostic accuracy was assumed to be around 93% for EUS-FNB combined with ROSE^[15–17]^ and around 93% for EUS-FNB combined with MOSE^[18]^, according to previous studies. If the noninferiority margin was set to 10%, the sample size would have to be 162 patients in each arm to achieve a statistical power of 80% within an error of 0.05. Assuming a 5% dropout rate, a final sample size of 170 (85 per group) was estimated to be required. Descriptive statistics are presented as the mean ± standard deviation (SD) values or the median and range values for continuous variables and as frequencies for categorical variables. The 2 groups were compared using analysis of variance for continuous variables, Kruskal-Wallis tests for numbers of events, and Pearson *χ*^2^ test or Fisher exact test for categorical variables. Differences with *P* < 0.05 were considered significant. All data were statistically analyzed using SPSS version 13.0 statistical software (SPSS, Chicago, IL).

## RESULTS

In this study, a total of 173 patients were enrolled. Of these patients, 2 were excluded because of a change of mind after being included in the study and because of perforation during scope insertion. Therefore, 171 patients were randomized, 85 to the ROSE group (median age, 72 years; 49 male and 36 female patients) and 86 to the MOSE group (median age, 73 years; 48 male and 38 female patients).

Table 1 shows the baseline demographic characteristics and results of the procedures. ECOG PS was mainly 0 or 1 in each group, with no significant difference (*P* = 0.6111). Pancreatic lesions were observed at the head/uncinate (n = 41), body (n = 26), and tail (n = 18) in the ROSE group and the head/uncinate (n = 42), body (n = 27), and tail (n = 17) in the MOSE group. There was no significant difference between the 2 groups (*P* = 0.9735). Mean tumor size was 22.8 mm in the ROSE group and 22.7 mm in the MOSE group (*P* = 0.9732). Laboratory data, such as the white blood cell and platelet counts and hemoglobin, were not significantly different between the 2 groups. Surgical resection was performed for 29 patients in the ROSE group and 27 patients in the MOSE group (*P* = 0.7461). Regarding the final diagnosis, pancreatic carcinoma was mainly observed in the ROSE group (72.9%, *n* = 62) and the MOSE group (76.7%, *n* = 66). The final diagnosis was not significantly different between the 2 groups (*P* = 0.9422). All patients successfully underwent EUS-FNB. The puncture sites in the ROSE and MOSE groups, respectively, were as follows: stomach (*n* = 43, *n* = 45); duodenal bulb (*n* = 17, *n* = 17; and second part of the duodenum (*n* = 25, *n* = 24) (*P* = 0.9704). The mean number of punctures was significantly lower in the MOSE group than in the ROSE group (1.47 *vs*. 1.20, *P* = 0.0171). The mean length of visible core tissue was 13.0 mm. Adverse events associated with the procedure were observed in 3 patients in the ROSE group (abscess of the stomach wall, *n* = 1; pancreatitis, *n* = 2) and in 1 patient in the MOSE group (pancreatitis, *n* = 1). The mean follow-up period was not significantly different between the ROSE (466 days) and MOSE groups (507 days).

**Table 1:** Patients’ characteristics and results of the procedures

Characteristics	ROSE	MOSE	*P*
Total patients, *n*	85	86	—
Median age, range, y	72 (38–91)	73 (44–89)	0.4057
Sex (male/female)	49/36	48/38	0.8089
ECOG PS			0.6111
0	48	44	
1	29	30	
2	16	24	
Pancreatic lesion site			0.9735
Head/uncinate	41	42	
Body	26	27	
Tail	18	17	
Tumor size, mean ± SD, mm	22.8 ± 8.75	22.7 ± 10.18	0.9732
Platelet count, mean ± SD, ×10^4^/μL	23.2 ± 7.38	23.0 ± 7.92	0.8790
Hemoglobin, mean ± SD, g/dL	12.7 ± 1.87	12.7 ± 1.58	0.9016
Surgical resection, *n*	29	27	0.7461
Final diagnosis, *n*			0.9422
Pancreatic adenocarcinoma	62	66	
Neuroendocrine tumor	4	4	
Autoimmune pancreatitis	4	6	
Metastatic pancreatic tumor	3	2	
Chronic pancreatitis	7	4	
Serous cyst neoplasm	2	2	
Other benign lesions	3	2	
Puncture rote			0.9704
Stomach	43	45	
Duodenal bulb	17	17	
Second part of the duodenum	25	24	
No. of punctures, mean (range)	1.47 (1–4)	1.20 (1–3)	0.0171
Length of macroscopic visible core tissue, mean (range), mm	—	13.0 (4–58)	—
Overall adverse events, *n*	3	1	0.3059
Follow-up period, mean, d	507	466	0.4549

Table 2 shows the results of ROSE during EUS-FNB. After the first passage, an adequate sample was obtained in 74.1% of patients (63/85). However, indeterminate (7.1%, 6/85) or insufficient (18.8%, 16/85) results were observed. An adequate sample was obtained in 40.1% (9/22) during the second passage, in 36.4% (4/11) during the third passage, and in 100% (7/7) during the fourth passage.

**Table 2:** Results of rapid on-site evaluation

	Adequate	Indeterminate	Insufficient
First pass, % (*n*)	74.1 (63/85)	7.1 (6/85)	18.8 (16/85)
Second pass, % (*n*)	40.1 (9/22)	18.8 (4/22)	31.8 (7/22)
Third pass, % (*n*)	36.4 (4/11)	54.5 (6/11)	9.1 (1/11)
Fourth pass, % (*n*)	100 (7/7)	—	—

Table 3 shows the results of MOSE during EUS-FNB. After the first passage, MOSE was positive in 84.9% of patients (73/85). Also, MOSE was positive in 69.2% (9/13) during the second passage and in 100% (4/4) during the third passage.

**Table 3:** Results of macroscopic evaluation

	MOSE (+)	Length of macroscopic visible core tissue, mean (range), mm	MOSE (−)
First pass, % (*n*)	84.9 (73/86)	11.3 (4–36)	15.1 (13/86)
Second pass, % (*n*)	69.2 (9/13)	28.1 (16–41)	30.8 (4/13)
Third pass, % (*n*)	100 (4/4)	11.5 (6–18)	—

Table 4 shows the results for the diagnostic yields of the ROSE and MOSE groups. In the ROSE group, diagnostic sensitivity and accuracy were 88.6% and 82.4%, respectively, for cytology and 94.3% and 91.8%, respectively, for histology. On the other hand, in the MOSE group, diagnostic sensitivity and accuracy were 94.4% and 91.8%, respectively, for visible core tissue and 80.6% and 70.0%, respectively, for red tissue. Finally, overall diagnostic sensitivity and accuracy were 97.1% and 95.3%, respectively, in the ROSE group and 95.8% and 95.3%, respectively, in the MOSE group. Therefore, the diagnostic yields of the ROSE and MOSE groups were similar. Finally, because the cost of ROSE was about $31 per one session, total cost was higher in ROSE, about $2635 compared with MOSE.

**Table 4:** Diagnostic yield

	Cytology (ROSE)	Histology (ROSE)	Overall (ROSE)	Visible core tissue (MOSE)	Red tissue (MOSE)	Overall (MOSE)
Sensitivity, %	88.6 (62/70)	94.3 (66/70)	97.1 (68/70)	94.4 (68/72)	80.6 (58/72)	95.8 (69/72)
Specificity, %	100 (15/15)	100 (15/15)	100 (15/15)	100 (14/14)	100 (14/14)	100 (14/14)
PPV, %	100 (62/62)	100 (66/66)	100 (68/68)	100 (68/68)	100 (58/58)	100 (69/69)
NPV, %	65.2 (15/23)	78.9 (15/19)	88. 2 (15/17)	77.8 (14/18)	50 (14/28)	82.4 (14/17)
Accuracy, %	82.4 (70/85)	91.8 (78/85)	95. 3 (80/85)	91.8 (79/86)	70.0 (60/86)	95.3 (82/86)

NPV, negative predictive value; PPV, positive predictive value.

## DISCUSSION

EUS-FNA and FNB are now established techniques to obtain cytological confirmation for the diagnosis of pancreatic lesions. To improve diagnostic yield, various efforts such as ROSE have been reported. According to a meta-analysis of EUS-FNA combined with ROSE that included 70 studies,^[4]^ ROSE was associated with a statistically significant improvement of up to 3.5% in the adequacy rate. In addition, according to the most recent study of ROSE,^[5]^ sensitivity increased from 63.7% to around 91%. Therefore, during EUS-FNA, ROSE might be useful to improve the diagnostic yield. However, if an on-site cytopathologist is not available, EUS-FNA combined with ROSE might be challenging. Indeed, according to an online questionnaire of the international endosonographic community, including 400 endosonographers from the United States, Europe, and Asia,^[5]^ ROSE was only available to 65% because of limited pathology staffing. On the other hand, MOSE can be performed without cytopathologists.^[17]^ Chong et al. conducted a randomized controlled trial of MOSE and ROSE during EUS-FNA for pancreatic lesions.^[18]^ The 244 patients were divided into ROSE and MOSE groups. They found that the diagnostic yield for MOSE (92.6%) was similar to that for ROSE (89.3%; *P* = 0.37); therefore, if ROSE is not available, MOSE may be one of the alternative techniques to improve diagnostic yield.

As other recent efforts to improve diagnostic yield, various FNB needles, which can obtain more tissue, such as reverse-bevel, Menghini-tip, Franseen, or fork-tip needles, have become available. Itonaga et al. compared the diagnostic yield of EUS-FNA using a 22-gauge standard needle and a Franseen needle for pancreatic lesions. In the 523 patients, compared with standard FNA (*n* = 260), diagnostic accuracy (71.2% *vs*. 84.0%) and sensitivity (66.7% *vs*. 82.4%) were significantly higher for the Franseen needle (*n* = 263). This result may be based on the amount of tissue obtained. Indeed, according to a randomized controlled trial of the tissue amounts of standard FNA and FNB,^[19]^ the mean total tissue area was significantly larger in FNB samples (2.74 mm^2^) than in FNA samples (0.44 mm^2^; *P* < 0.001). In addition, mean diagnostic tissue area was significantly larger in the FNB samples (1.74 mm^2^) than in the FNA samples (0.28 mm^2^; *P* < 0.001). Therefore, because the rate of FNB needle use has increased in recent years, the role of ROSE may be unclear. Crinò et al. conducted a randomized controlled noninferiority trial between EUS-FNB with and without ROSE.^[20]^ This study included 800 patients (385 with ROSE and 386 without). The noninferiority of EUS-FNB without ROSE was confirmed, with an absolute risk difference of 1.0% (1-sided 90% confidence interval, −1.1% to 3.1%; noninferiority *P* < 0.001), although the sample quality of histological specimens and diagnostic yield such as sensitivity and accuracy were similar. The rate of obtaining core tissue was significantly higher in FNB without ROSE than with FNB with ROSE (78.0% *vs*. 70.7%, *P* = 0.021). In addition, according to a recent meta-analysis including 8 studies with 2147 patients,^[21]^ sample adequacy was not significantly different between FNB with (95.5%) and without ROSE (88.9%; *P* = 0.07), but the diagnostic accuracy was significantly higher for EUS-FNB with ROSE than for FNB alone (OR = 0.249, 1.19–5.73; *P* = 0.03). Therefore, according to previous reports, the benefits of ROSE during EUS-FNB might still be unclear. Previous studies may have several limitations. First, the size of the needle was not the same. Second, several kinds of needles were used. In addition, after puncturing, various techniques, such as suction, wet suction, or the slow-pull technique, were attempted. These might influence the amount of tissue obtained or the diagnostic yield. According to a comparison study between needles and methods of EUS-FNB,^[22]^ cellularity of obtained tissue was significantly higher by EUS-FNB using fork-tip or Franseen needles than with reverse-bevel or Menghini-tip needles (*P* < 0.001). Furthermore, tumor tissue was identified with greater than 90% accuracy in samples of EUS-FNB using fork-tip or Franseen needles compared with the others (*P* < 0.001). Moreover, diagnostic yield differed for no suction, slow-pull, or suction techniques among the needles. Therefore, to evaluate the true effectiveness of ROSE, it might be important to standardize the kind of needle or method of EUS-FNB. On the other hand, the present randomized controlled trial was conducted using the same FNB needle (fork-tip) and same FNB method (slow-pull). Therefore, in our view, although our study was similar to previous studies and meta-analysis and might not add new knowledge, the present study might have been able to evaluate whether ROSE was useful. According to the present study, ROSE did not improve diagnostic yield and increased the number of punctures, although adverse events were not significantly different between the 2 groups. Also, as assessing patient-centered outcomes, all patients were not complicated with pain associated with procedure, except 2 patients who were complicated with pancreatitis. Discomfort was not observed in all patients. There was no significant difference in the diagnostic yield between the 2 groups. In addition, during procedure, sedation was used. Therefore, we assumed that patient’s satisfaction might not be different, although we could not record.

There were several limitations to our study. First, although the present study was a multicenter, randomized controlled trial, only Japanese centers were included. Second, only experienced, high-volume centers were included, and therefore, the present results might not be reproducible in other settings. Third, our study was not blinding of the endoscopists, which could introduce performance bias, particularly in subjective outcomes such as the number of needle passes and tissue adequacy. In addition, the sample size was focused on the primary outcome; it might have been underpowered to detect differences in secondary outcomes such as adverse events. Finally, cytopathological evaluation was not performed centrally, and diagnostic ability might not have been the same across the 3 centers. Therefore, although the present study might be the first study of ROSE evaluation using the same needle and the same method, further randomized controlled studies in an international setting, with central cytopathological evaluation and including nonexpert endosonographers, are needed.

In conclusion, EUS-FNB using a fork-tip needle for pancreatic lesions has high diagnostic accuracy even without ROSE comparable to EUS-FNB with ROSE.

## Acknowledgments

None.

## Declaration of Patient Consent

Informed consent was obtained from the patient for the publication of their information and imaging.

## Source of Funding

None.

## Ethical Approval

This trial was approved by the Certified Review Board of Osaka Medical and Pharmaceutical University Hospital (2020-015-4). Name of clinical registry and number: The University Hospital Medical Information Network Clinical Trials Registry (UMIN000041676).

## Conflict of Interest

The authors declare that they have no financial conflict of interest with regard to the content of this report.
